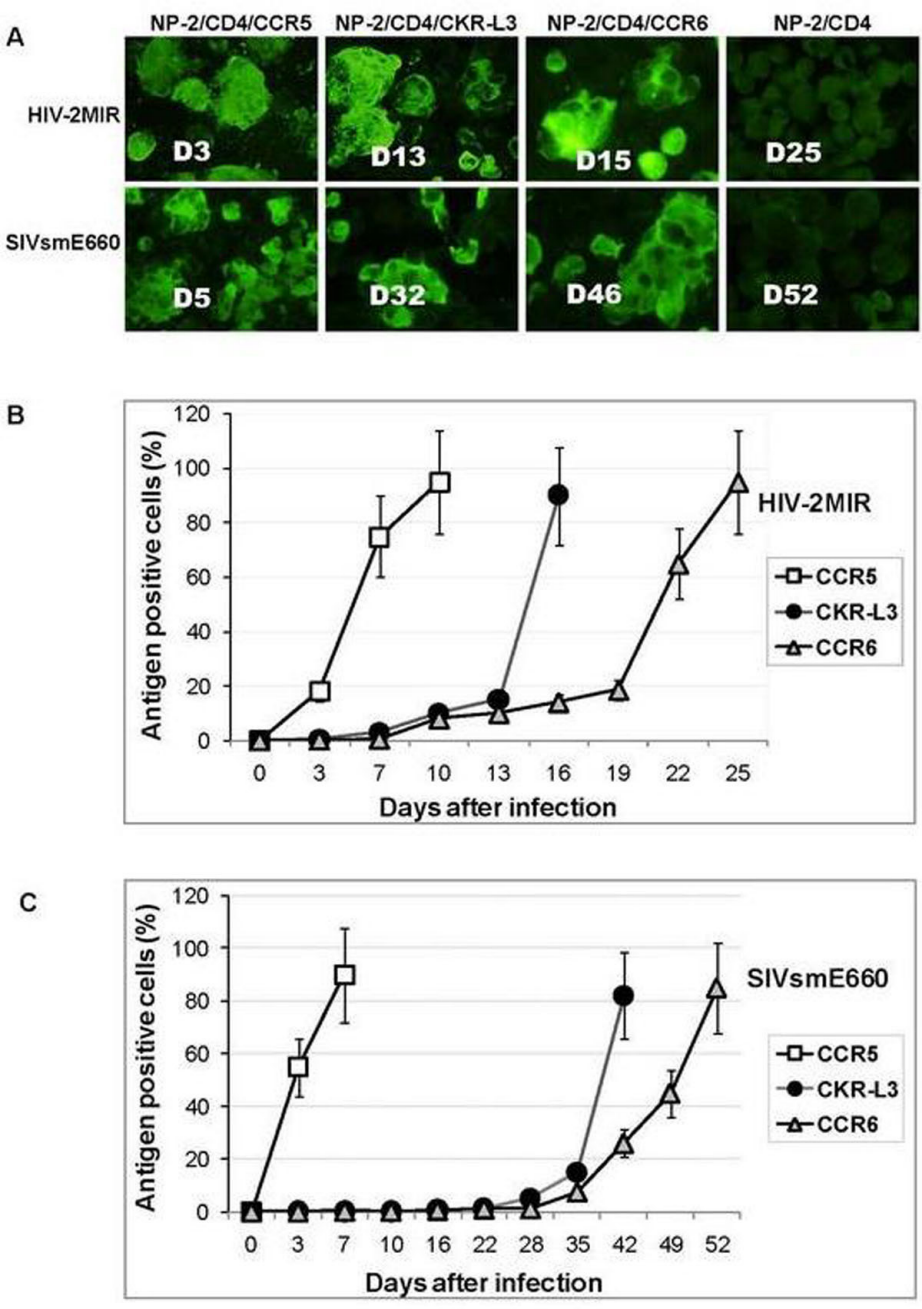# CKR-L3, a deletion version CCR6-isoform shows coreceptor-activity for limited human and simian immunodeficiency viruses

**DOI:** 10.1186/1471-2334-14-S2-P69

**Published:** 2014-05-23

**Authors:** Salequl Islam, Nobuaki Shimizu, Takahiro Ohtsuki, Atsushi Jinno-Oue, Atsushi Tanaka, Hiroo Hoshino

**Affiliations:** 1 Johns Hopkins University, Baltimore, USA

## Background

Chemokine receptors (CKRs), CCR5 and CXCR4 function as major coreceptors in human/simian immunodeficiency virus (HIV/SIV) infections. About 20 alternative G protein-coupled receptors (GPCRs) have been identified as minor coreceptors for the viruses. We reported CCR6 as an alternative coreceptor. A five-amino acid shorter isoform of CCR6, namely CKR-L3, was examined for its coreceptor function and described in this report.

## Methods

NP-2 cells transduced with CD4-receptor (NP-2/CD4) normally remain resistant to all HIV/SIV infection; however, further introduction of functional coreceptor can make the cells susceptible to the viruses. NP-2/CD4/CKR-L3 cells were produced to examine coreceptor activity of CKR-L3. Viral antigen in infected NP-2/CD4/coreceptor cells was detected by indirect immunofluorescence assay (IFA). The results were validated by detection of syncytia, proviral DNA and by measuring reverse transcriptase (RT) activities.

## Results

HIV-2MIR and SIVsmE660 were found to infect NP-2/CD4/CKR-L3 cells. This justifies the coreceptor function of CKR-L3. Viral antigens appeared faster in NP-2/CD4/CKR-L3 cells than in NP-2/CD4/CCR6, indicates that the CKR-L3 carries more efficient coreceptor-activity. Moreover, syncytia formation was sooner, RT release was higher and earlier through CKR-L3 compared to CCR6. Partial sequence analyses of HIV-2MIR and SIVsmE660 replicated through CKR-L3 and CCR6 coreceptor showed some divergence in envelope region compared to the parental CCR5-variant.

## Conclusions

Isoform CKR-L3 exhibited coreceptor activity for limited primary HIV/SIV isolates with better efficiency than CCR6-isoform. Amino acid substitutions in the envelope region of the viruses may confer selective pressure towards CKR-L3-use. CKR-L3 with other minor coreceptors may contribute to HIV/SIV pathogenesis including dissemination, trafficking and latency.

**Figure 1:**